# The Rhythm of Connection: Describing the Heartbeats Intervention for Patients and Families Receiving Paediatric Palliative Care

**DOI:** 10.3390/children12070884

**Published:** 2025-07-03

**Authors:** Holly E. Evans, Matthew Ralph, Tiina Jaaniste, Claire E. Wakefield, Ursula M. Sansom-Daly

**Affiliations:** 1 Behavioural Sciences Unit, School of Clinical Medicine, UNSW Medicine and Health, Randwick Clinical Campus, Discipline of Paediatrics and Child Health, University of New South Wales Sydney, Kensington, NSW 2031, Australia; c.wakefield@unsw.edu.au (C.E.W.); ursula@unsw.edu.au (U.M.S.-D.); 2 Kids Cancer Centre, Sydney Children’s Hospital, Randwick, NSW 2031, Australia; 3 Department of Palliative Care, Sydney Children’s Hospital, Randwick, NSW 2031, Australia; matt.ralph@health.nsw.gov.au; 4 School of Clinical Medicine, Discipline of Paediatrics and Child Health, University of New South Wales Sydney, Kensington, NSW 2031, Australia; tiina.jaaniste@health.nsw.gov.au; 5 Division of Quality of Life and Pediatric Palliative Care, Department of Pediatrics, Stanford University and Stanford Medicine Children’s Health, Palo Alto, CA 94305, USA; 6 Sydney Youth Cancer Service, Nelune Comprehensive Cancer Centre, Prince of Wales Hospital, Randwick, NSW 2031, Australia

**Keywords:** paediatric palliative care, music therapy, heartbeat recordings, integrative medicine, children and adolescents, cancer

## Abstract

Music therapy in paediatric palliative care offers a unique opportunity for emotional support, legacy creation, and therapeutic connection for children and their families. This paper describes the Heartbeats Intervention, as delivered by a paediatric palliative care music therapist at Sydney Children’s Hospital Australia. This intervention involves recording and creatively integrating the heartbeats of children and family members into personalised musical compositions. Originally inspired by music therapist Brian Schreck’s work, the intervention has evolved to meet diverse therapeutic goals, from soothing children with serious illnesses (including cancer) with recordings of their families’ heartbeats to creating legacy song tracks that support families through bereavement. Despite some logistical and resource challenges, the intervention has been well-received and continues to expand, including the integration of environmental soundscapes and broader community involvement, which allows the intervention to be experienced by a greater number of families. This paper contributes to the limited but growing literature on music therapy in paediatric palliative care, highlighting the Heartbeats Intervention as a flexible and meaningful way to enhance psychosocial support and connection for children and their families. Further research to evaluate its long-term impact and to explore children’s direct experiences of the intervention is needed.

## 1. Introduction

Music therapy in paediatric palliative care offers an opportunity to support children and families through some of the most challenging moments of their journey. For hospitalised children who have experienced profound disruption to their lives, music therapy can provide important opportunities for “normal” childhood experiences, as well as allowing choice and control in a developmentally appropriate way [1]. Within the paediatric palliative care context, music therapy is facilitated by qualified music therapists and may involve children and families listening to music or rhythm and playing or composing music. Music therapy may be useful in promoting quality of life, wellbeing, and positive emotions in paediatric patients [2]. Benefits may also extend beyond the child themselves and to the whole family system [3] by creating space for positive interactions, which build relationships and family wellbeing, as well as in supporting families through bereavement [4,5].

## 2. The Use of Heartbeat Recordings as a Music Therapy Intervention

Music therapist Brian Schreck developed the amplified cardiopulmonary recordings intervention, often called “heartbeat recordings” at Cincinnati Children’s Hospital Medical Centre, USA. Heartbeats have natural rhythm and vibrational qualities which can be recorded and experienced on their own or as part of a soundtrack with additional elements like music or voice, while having relational significance in that closeness is ordinarily required to hear others’ heartbeats. Schreck first published about the intervention in 2013, describing the use of the intervention in perinatal hospice care [6,7], and later paediatric palliative care for children with progressive neurodegenerative illnesses [8]. The intervention is now delivered in various forms, both in paediatric and adult care, in more than 80 medical centres in the USA [9].

The intervention has been described and evaluated for use in a neonatal intensive care setting, with small-scale survey data indicating clinical safety and acceptability of the intervention for these families [10,11]. Similar intervention studies have found that playing maternal heartbeat sounds for neonates in intensive care improved short term physiological parameters such as respiratory rates and oxygen saturation, and increased weight gain in the longer term [12,13]. In this context, complex music may be overstimulating for neonates, but the simplicity of rhythmic heartbeat and sounds may be beneficial [14]. Theoretically, this may also be the case for older seriously unwell children; however, to date, studies have not yet explored whether simple heartbeat sounds could also benefit this group.

Heartbeat recordings have also been researched in the bereavement setting. Walden et al. [8] describe a qualitative study of a heartbeat intervention with 11 bereaved families of children with progressive neurodegenerative illnesses. Parents were interviewed three months after receiving their child’s heartbeat song (a heartbeat embedded in a musical composition). Qualitative themes highlighted parents’ experiences of grief and sorrow, but also the potential for the heartbeat recordings to facilitate connection with their child through tangible legacy creation. Participants’ statements revealed differences between families in the importance or purpose they ascribed to the recordings, with some emphasising the significance of the heartbeat itself and others sharing that the heartbeat alone without the song lacked some meaning offered by combining the heartbeat with a song. This underscores the importance of flexibility for music therapists in responding to family wishes and intended purpose for the recordings.

Ghetti et al. [15] have recently reported on an action research single case study in which a child’s heartbeat recordings were used in music therapy bereavement support of a father. Although caution must be exercised in drawing conclusions from a single case, Ghetti et al. [15] described the heartbeat recording intervention as a tool for fostering resilience and positive growth in a grieving father. Indeed, in the context of bereavement care, the adaptability of the intervention meets families’ needs for flexible approaches which are often not able to be delivered as part of traditional hospital-based bereavement support [16].

Although these early data are promising, there is a lack of available information on how the intervention is being applied in different medical settings and clinical contexts. This paper therefore describes and discusses how the paediatric palliative care programme at Sydney Children’s Hospital Australia delivers a heartbeats intervention, with the aim of contributing to the broader literature on music therapy interventions for this special population.

## 3. The Heartbeats Intervention at Sydney Children’s Hospital

The Heartbeats Intervention has been provided as part of the music therapy programme at Sydney Children’s Hospital since 2018 and involves recording children’s and family members’ heartbeats to be used in various creative ways to meet therapeutic goals, depending on the wishes of the family. It is based on the process-based intervention developed by Brian Schreck in 2013 and generously shared with our team [6].

The use of the intervention in our centre varies from family to family. Initially, we used the Heartbeats Intervention primarily as a legacy and memory-making intervention in the end-of-life setting for families. However, recently our music therapy and palliative care teams have first introduced families to the idea of heartbeat recordings by offering to record parent or family members’ heartbeats as a means to comfort and sooth unwell children at earlier stages in their care journey. For this purpose, a small speaker can be placed inside a teddy or other toy, which has a soft vibrational quality when playing heartbeat sounds only. At Sydney Children’s Hospital, we offer the Heartbeats Intervention to approximately one family per week.

There are many ways that a family may wish to connect with the heartbeat recordings (both of parents and their children’s), and facilitators work with families to realise this vision for them. Once a child’s heartbeat is recorded, it can be utilised in many creative ways, which may not be seen solely as legacy-making for children who are approaching end-of-life. If the heartbeat is used at a later time in bereavement, then the team discusses this further with families to decide whether they would like a special song or instrumental backing to accompany the heartbeat recording, often creating an original musical track of three to four minutes. We have been able to use both original music written by the music therapist, and as well as recorded “cover” versions of existing songs that families request.

**Figure 1:**
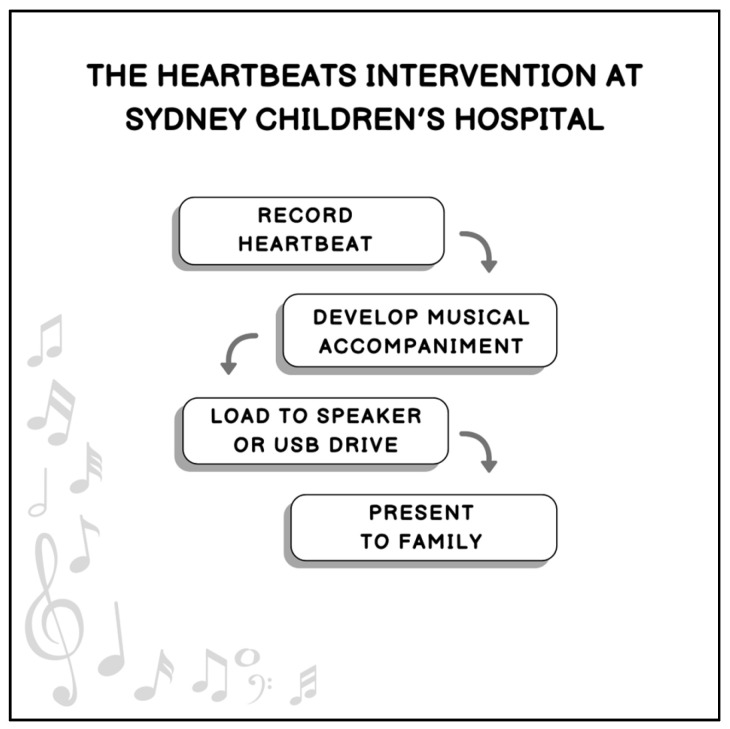
Steps in the Heartbeats Intervention.

To date, our team has been able to use the intervention in acute situations (e.g., neonatal intensive care or childhood accidents) as well as in traditional palliative care settings, where the medical and music therapy teams have known the family for some time and have built rapport. Discussions with families and the progression of developing heartbeat music recordings are essentially led by when the family feels ready to discuss their wishes. As such, open communication with the wider palliative care team and the family plays an important role in delivering this service and creating the recording. Figure 1 outlines the steps in the Heartbeats Intervention at Sydney Children’s Hospital.

## 4. The Recording Process–Practical Aspects

### 4.1. Heartbeat Recording

To record heartbeats at Sydney Children’s Hospital, we use a Stethsman Littman Bluetooth enabled stethoscope (approximately 600 AUD). This stethoscope can save up to 15 recordings on the device or can be connected wirelessly to directly transfer recordings onto a laptop. The stethoscope can filter out most extraneous noise; however, some medical devices (e.g., ventilators) can cause interference. This can be circumvented by capturing small sections of heartbeat in between mechanical noises to then cut together and loop. In time-sensitive situations where a music therapist is not available to do the recording, we have included instructions written inside the stethoscope box so that other team members (e.g., nurses) can complete the recording. At least one minute of heartbeat recording is ideal to allow the final audio to be seamlessly looped together, matching accents and waveforms which can vary between individuals. For a 30 min vibrational experience of family member’s heartbeats for patients, longer recordings reduce the number of transitions or loops required. In total, our team spends approximately 30 to 45 min with the family to complete these initial recordings.

Once the heartbeats have been recorded, the music therapist is able to mix and loop the heartbeat recording. Logic Pro software Version 11 (at cost) is used for looping the heartbeat recording as well as recording and mixing the instruments to accompany the heartbeat. Skill in using the software is required to make looping seamless and to match rhythms, as well as shaping the frequency range to increase the vibration of the speaker for heartbeat only recordings in soft toys. It can take the music therapist 45 min to an hour to loop the heartbeat recording and export. We send the heartbeat recording to families as soon as possible after recording to maximise their ability to use the recording with their child.

### 4.2. Development of Musical Accompaniment

If requested by the family, our team will then record a musical accompaniment to the heartbeat. Royalty-free prerecorded tracks can be used; however, our team will often record original music, or covers of songs requested by families. Having a quiet space to record the music is essential, as listening to the recording is a very focused and intimate experience and added exterior or unrelated sounds can be disruptive. At Sydney Children’s Hospital, we use on-site audiology rooms, which while purpose-built for other functions, are soundproofed and provide an isolated environment for soft and delicate guitar accompaniment. Development of musical backing can be in collaboration with families and an iterative process.

### 4.3. Presentation to Families

Once the recording is completed, we can provide the recording on a Bluetooth speaker which is given to the families as a soothing device for their child. Sometimes, this speaker is placed in a soft toy or cushion. In this context, one of the parents’ heartbeats is looped into a 30 min recording which enables a continuous vibrational experience for the child. In practice, this can help to ease into recording the child’s heartbeat as a legacy-making aspect of the intervention.

We also transfer recordings of the heartbeat both with and without accompaniment to a USB drive and present this to families in a small wooden case created by the Shire Wood Working Club (New South Wales, Australia). The highest quality files with music can be up to 256 MB, and as such are too large to email. There are occasions where several USB drives and cases are provided to meet the needs of the family.

In the event of the child’s death, the heartbeat recording set to music can become a space of intimate connection with the child for bereaved families, framed within a three-minute track. The USB drives and cases can also become physical keepsakes for the family.

### 4.4. Adaptations and New Developments of the Intervention, Including Community Involvement

The intervention has received positive feedback from families, and the team experiences ongoing demand. Recently, there have been opportunities to engage further with the local community to support the intervention and different ways in which it can be implemented/used/applied. For example, an increase in funding has allowed the music therapy department to purchase high quality teddy bears and speakers from a local supplier, and hospital volunteers will be engaged in installing zippers to the teddies for easy speaker insertion/charging. These teddy bears have also been used to provide recordings of environmental soundtracks and soundscapes, with and without the use of heartbeats, incorporating family members (including grandparents or cousins) saying comforting words. For some children, it has become a daily ritual for them to be greeted by their family member’s voice in the morning. We have also used the teddy bears with speakers to play parents’ heartbeats as a soothing and calming device for outpatient children undergoing painful or distressing procedures while in hospital.

## 5. Discussion

Overall, while there is a lack of systematic measurement of outcomes from heartbeat interventions, previous qualitative studies and our clinical observations suggest that the intervention is a positive and supportive experience for families. As there is limited research on music therapy for paediatric palliative care, there are few well-formed analyses of the applicable theoretical approaches to music therapy in this population to explain why particular interventions may be helpful in this setting. Indeed, music therapy interventions can be difficult to describe and operationalise, partly due to the complexity of musical stimuli [17].

However, the Heartbeats Intervention applies across the spectrum of music therapy, including receptive methods (e.g., listening) to active methods (e.g., instrument playing, including improvisation and songwriting). Theoretically, these aspects in combination can be used to approach individualised therapeutic goals in the immediate term related to symptom reduction, improved emotional states and relaxation. In the long-term, the intervention can provide opportunities for self-expression, choice-making, and externalising thoughts and emotions [18]. The Heartbeats Intervention can also function as a component of bereavement care. For example, the music therapist is able to flexibly continue supportive relationships with bereaved families through further refinement of music tracks and additional applications of heartbeat recordings at families’ request. The intervention also aligns with the continuing bonds theory of bereavement [19], which characterises the experience of continuing bonds to the deceased as psychologically helpful. Heartbeat recordings and accompanying music may support families’ continuing bonds with their child after death, creating a tangible and lasting legacy. The dual process model of grief [20,21] is also relevant; according to this model, people will naturally vacillate between loss and restoration-oriented states. The Heartbeats Intervention may be a meaningful and tangible way that families can sit with their grief when they are in a loss-focused state.

### 5.1. Challenges and Opportunities

Delivery of the Heartbeats intervention requires a confluence of skills on the music therapists’ part, both musical and technical skills, as well as creative and therapeutic skills. The success of the intervention can rely on the nuance of conversation as well as nuance in instrumentation and of mixing the heartbeat sounds. As with many aspects of palliative care, identifying the appropriate time to introduce the intervention can be challenging. It is often suggested close to a patient’s end of life, especially when the palliative care team has not had prior involvement with the family. It is often suggested close to a patient’s end-of-life. Sensitive communication skills are required, and often on the part of the whole team, as the music therapist may not be the only clinician introducing the intervention.

The intervention can be quite expensive to set up and deliver. There is an initial outlay for resources that are used on an ongoing basis (e.g., heartbeat recording equipment, mixing software) as well as consumables (speakers, USB drives, presentation boxes, soft toys). The quality of consumables is also important; a high-quality speaker is required for use in soft toys, as the sound quality needs to allow for vibration to be felt, but it also must be small enough to be inserted unobtrusively into a soft toy. For the intervention to be able to be delivered smoothly and effectively, the hospital or healthcare team must be able to maintain a supply of these consumables to quickly to respond to the needs of the patient and their family.

The intervention is relatively time-intensive compared to other forms of music therapy, where the technical and clinical activities are relatively contained. However, in spending more time with children and families to develop rapport and therapeutic relationships, it can become an important and meaningful way for music therapy to be involved in the support of patients in hospital, and later in bereavement support. Families are often touched that the palliative care team would invest in them, in both a financial and clinical way. However, the confluence of the above challenges can lead to inequities in access to the intervention. Access to palliative care for families, timing of availability of staff to record heartbeats (as well as resourcing constraints on having music therapy available in general) can mean that some families who would value it will not be able to receive the intervention.

Despite these challenges, in the palliative care setting, the Heartbeats Intervention can open the door to other conversations around goals of care and advance care planning. The intervention allows the music therapist to build relationships with children and families, and to earn their trust by being involved with something precious to them. It provides an opportunity for the multidisciplinary team to show care and support to the family, to acknowledge their pain and grief, and to facilitate palliative care “wrapping around” the family. The layers of support and connection around the family can even be experienced as extending into the wider community. In our team’s experience, it has been especially meaningful to involve members of the community directly in practical aspects of the intervention (for example, as volunteers sewing zippers into teddy bears). During the difficult period when a child is seriously unwell or dying, having tangible, meaningful ways for the community to contribute to caring for children in hospital allows families to feel that not only are they receiving professional and clinical support but also support from the wider community.

### 5.2. Future Directions

Little research, to date, has focused on music therapy in a paediatric palliative care setting [3]. Early research, however, shows that families and multidisciplinary teams value its ability to support child and family wellbeing. Particularly, a recent qualitative evidence synthesis [3] has emphasised the role of music therapy in offering paediatric palliative care patients emotional and physical reprieve, normalised childhood experiences, as well as strengthening family bonds and providing opportunities for quality time together. The accessibility of music therapy for children receiving palliative care is dependent upon the therapist’s ability to adapt to individual needs and the flexibility of their therapeutic work. However, even with skilled and flexible therapists, it seems likely that currently only a subset of children and families gain access to this kind of intervention. Data from Weaver and colleagues examining the structure of paediatric palliative care services (focusing on the USA but with some data from Canada and Europe) found that only 37% of healthcare professional respondents reported that music therapy was available and ‘often used’ [22]. This service access gap may be pronounced for older children: more recent data from our team looking at palliative care services for adolescents and young adults with cancer found that just 9% of our healthcare professional sample reported that music therapy services were available and ‘often used’ [23]. More work is required to ensure equity of access to music therapy in paediatric palliative care.

The therapeutic relationship is central to the success of music therapy, pointing to the importance of adequate and ongoing training and professional support of music therapists in the often-challenging paediatric palliative care setting. The Heartbeats intervention offers the flexibility and potential for ongoing therapeutic relationships from diagnosis through to bereavement which may make this a particularly powerful method of delivering music therapy for paediatric palliative care patients and families. However, further research is required to evaluate the intervention and fully explore the potential applications and strengths of heartbeat recordings, including identifying patients and families who might have most to gain from the intervention, finding out how to maximise positive outcomes, and examining the potential long-term supportive impact of heartbeat recordings, particularly for family members in bereavement. In general, music therapy research in this context would also benefit from a greater focus on children’s direct experiences of the intervention.

## 6. Conclusions

The Heartbeats Intervention allows healthcare to exist in a particularly sensitive space which is respectful of families’ needs and preferences and can offer families a lasting legacy for their child. While this is particularly significant in bereavement, the intervention is also of value for comforting unwell children and increasing their wellbeing. Further work is needed to fully understand its contribution to the lives of unwell children and their families. The intervention is an affirmation of a child and family experiencing life, measured by a heartbeat.

## Data Availability

No new data were created or analyzed in this study.
